# Recent Advances and Perspectives on Expanding the Chemical Diversity of Lasso Peptides

**DOI:** 10.3389/fbioe.2021.741364

**Published:** 2021-09-24

**Authors:** Mengjiao Wang, Christopher D. Fage, Yile He, Jinhui Mi, Yang Yang, Fei Li, Xiaoping An, Huahao Fan, Lihua Song, Shaozhou Zhu, Yigang Tong

**Affiliations:** ^1^ College of Life Science and Technology, Beijing University of Chemical Technology, Beijing, China; ^2^ Department of Chemistry, University of Warwick, Coventry, United Kingdom; ^3^ Clinical Laboratory Center, Taian City Central Hospital, Taian, China

**Keywords:** lasso peptide, biosynthesis, tailoring enzymes, natural products, post-translational modification, synthetic biology

## Abstract

Ribosomally synthesized and post-translationally modified peptides (RiPPs) are a growing family of natural products that exhibit a range of structures and bioactivities. Initially assembled from the twenty proteinogenic amino acids in a ribosome-dependent manner, RiPPs assume their peculiar bioactive structures through various post-translational modifications. The essential modifications representative of each subfamily of RiPP are performed on a precursor peptide by the so-called processing enzymes; however, various tailoring enzymes can also embellish the precursor peptide or processed peptide with additional functional groups. Lasso peptides are an interesting subfamily of RiPPs characterized by their unique lariat knot-like structure, wherein the C-terminal tail is inserted through a macrolactam ring fused by an isopeptide bond between the N-terminal amino group and an acidic side chain. Until recently, relatively few lasso peptides were found to be tailored with extra functional groups. Nevertheless, the development of new routes to diversify lasso peptides and thus introduce novel or enhanced biological, medicinally relevant, or catalytic properties is appealing. In this review, we highlight several strategies through which lasso peptides have been successfully modified and provide a brief overview of the latest findings on the tailoring of these peptides. We also propose future directions for lasso peptide tailoring as well as potential applications for these peptides in hybrid catalyst design.

## Introduction

Peptide natural products are one of the richest sources of biologically active compounds and are derived from numerous natural sources (Dang and Süssmuth, 2017). In particular, microbes produce peptide-derived secondary metabolites that exhibit a wide range of activities (Demain, 1999), including compounds that function in quorum-sensing (e.g., N-acyl homoserine lactones), agents that bind and transport metals (e.g., siderophores like enterobactin), and antibiotics that exert a survival advantage against other microbes (e.g., vancomycin) (Williams et al., 1989). Antibiotic resistance has outpaced the discovery of new antibiotics for years; antimicrobial peptides produced by microorganisms have thus attracted considerable attention as promising alternatives to the currently limited antibiotic pipeline (Neu, 1992; Newman and Cragg, 2020; Al Musaimi et al., 2021). Generally, peptide natural products can be classified into several families based on their biosynthetic machinery. Members of one such family are synthesized by megadalton, multi-modular enzymatic assembly lines known as nonribosomal peptide synthetases (NRPSs), which produce structurally diverse peptides in a ribosome-independent manner (Süssmuth and Mainz, 2017). Another family consists of assorted cyclic dipeptides assembled by tRNA-dependent cyclodipeptide synthases (Belin et al., 2012). Yet another family, and the subject of this review, is comprised of the ribosomally synthesized and post-translationally modified peptides (RiPPs), which employ processing and tailoring enzymes to generate a wide variety of structures and functions that are inaccessible to most peptides of ribosomal original (Arnison et al., 2013).

**FIGURE 1:**
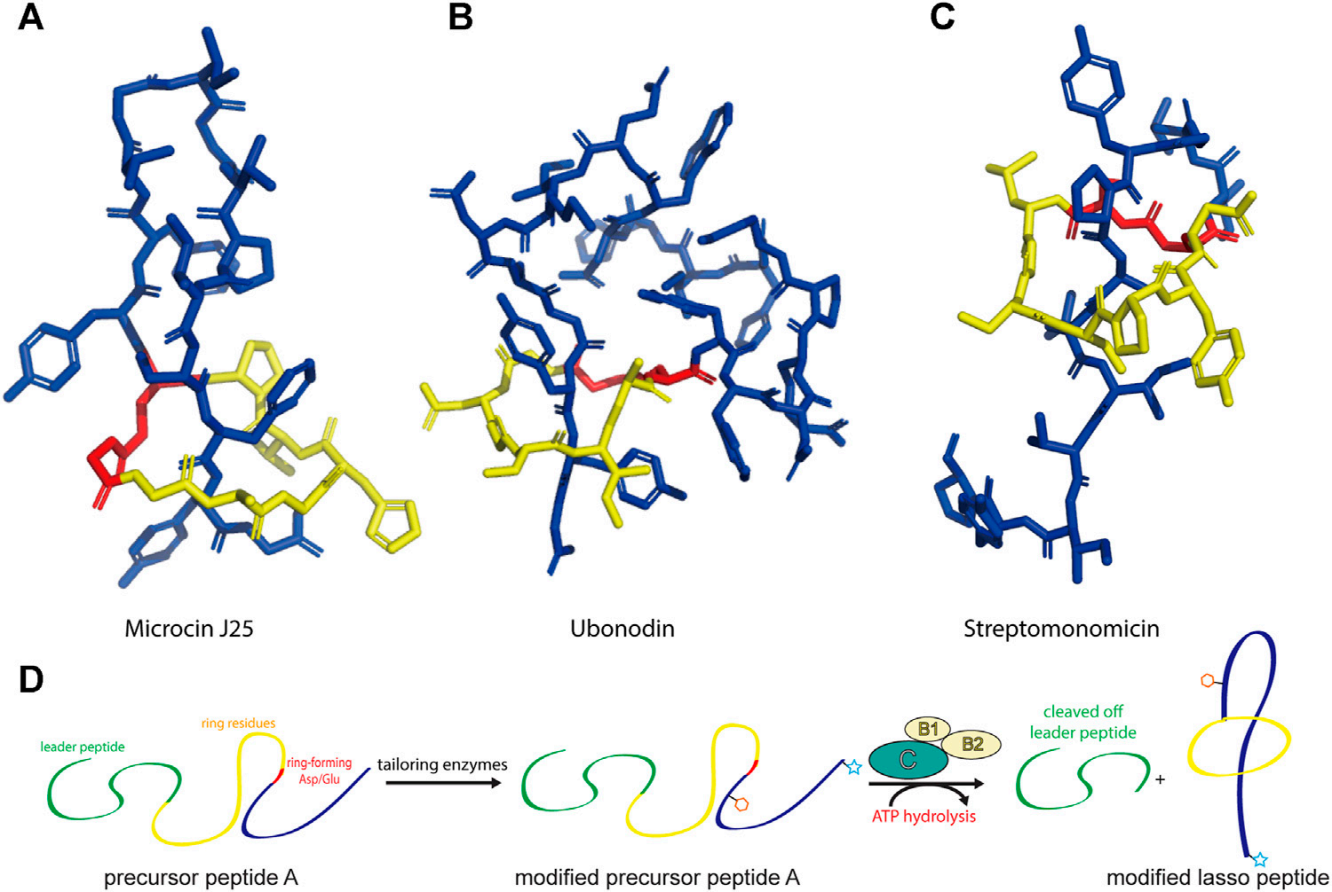
Representative structures of lasso peptides: **(A)** microcin J25 (PDB code 1Q71) (Rosengren et al., 2003), **(B)** ubonodin (PDB code 6POR) (Cheung-Lee et al., 2020), and **(C)** streptomonomicin (PDB code 2MW3) (Metelev et al., 2015). The ring is highlighted in yellow, the tail in blue, and the ring-forming Asp/Glu in red. **(D)** Schematic of the suggested pathway for modified lasso peptide biosynthesis.

Lasso peptides are an expanding subfamily of RiPPs endowed with fascinating properties (Hegemann et al., 2015). As the name suggests, they adopt a lariat knot-like fold in which the C-terminal tail is threaded through a 7–9-residue macrolactam ring formed between the N-terminal amino group and the carboxy side chain of an Asp or Glu residue. This entropically disfavored conformation is stabilized by disulfide bonds and/or bulky plug residues above/below the ring that secure the threaded C-terminus (Figure 1). Due to their unique topology, lasso peptides are often resistant to heat and chemical denaturation as well as proteolytic degradation, and they possess valuable bioactivities, such as antimicrobial, antiviral, enzyme inhibitory, receptor antagonist, and antitumor activities (Maksimov et al., 2012; Hegemann et al., 2015; Cheng and Hua, 2020).

Since the discovery of the biosynthetic pathway for microcin J25, the general principles of lasso peptide assembly have been steadily delineated (Duquesne et al., 2007). Although more than 10 different clades of lasso peptide biosynthetic gene clusters have been reported, in all cases the precursor peptide (“A”) is matured through the activity of the processing enzymes: a peptidase known as the B protein, which cleaves the leader peptide, and an ATP-dependent cyclase known as the C protein, which catalyzes macrolactam formation on the core peptide (Figure 1D). In several biosynthetic gene clusters, the B protein is divided into smaller B1 and B2 fragments that share homology with PqqD and transglutaminase domains, respectively (Maksimov et al., 2012; Hegemann et al., 2018; Chekan et al., 2019; DiCaprio et al., 2019). The B1 protein adopts a conserved function across all RiPP families, serving as a molecular chaperone that binds the leader peptide and delivers the precursor peptide to the B2 protein for leader peptide cleavage (Maksimov and Link, 2014; Burkhart et al., 2015; Zhu et al., 2016a; Koos and Link, 2019; Sumida et al., 2019).

Most of the reported lasso peptides possess the eponymous knotted structure. Other than this topology, few post-translational modifications (PTMs) of lasso peptides have been described until recently, perhaps be due to bias in selecting organisms and/or clusters for study (Zhu et al., 2016c). Nevertheless, the development of versatile tools to engineer lasso peptides with altered biological activities or physicochemical properties has garnered attention from pharmaceutical chemists and synthetic biologists alike. With the advent of new technologies and a better understanding of lasso peptide biosynthesis over the last decade, the goal of lasso peptide engineering has become more achievable.

In the current review, we describe recent developments in synthetic biology strategies towards the preparation of new lasso peptide derivatives. Recent advances in the characterization of various tailoring enzymes that diversify lasso peptides are also summarized, and future directions for lasso peptide engineering are proposed.

### Improvement and Expansion of Lasso Peptide Function by Mutagenesis

Compared to peptides assembled by megasynthetases (i.e., NRPSs), the ribosomal origin of RiPPs allows for significant changes to their chemical structures via site-directed mutagenesis of precursor peptides (Truman, 2016). Normally, the processing enzymes involved in a RiPP pathway specifically recognize the leader peptide and exhibit remarkable substrate tolerance for different core peptides (Oman and van der Donk, 2010). From a biotechnological perspective, this offers an excellent opportunity to expand the chemical functionality of these peptides at a low genetic cost (Urban et al., 2017; Hetrick et al., 2018; Yang et al., 2018; Hegemann et al., 2019; Si et al., 2021). Thus, mutagenesis is an effective tool for the generation of RiPP libraries and is the simplest strategy for diversifying the structures of peptide natural products in a predictable and rapid manner (Elsayed et al., 2015).

To date, several examples of RiPPs, including lasso peptides, have been engineered in this manner. For example, newly identified lasso peptides such as capistruin, astexin, caulosegnin, and paeninodin have been extensively studied through mutagenesis experiments (Knappe et al., 2009; Hegemann et al., 2013a; Li et al., 2015). The structure-activity relationship of the anti-mycobacterial lasso peptide lariatin A was also investigated through mutational analysis, revealing crucial roles for Tyr6, Gly11, and Asn14 in the compound’s antibacterial activity, and essential roles for Val15, Ile16, and Pro18 in enhancing that activity (Inokoshi et al., 2016). Several point mutants of lariatin A showed improved anti-mycobacterial activity relative to the wild-type sequence, suggesting that genetic engineering is a promising method for the efficient design of lariatin analogs to counter tuberculosis (Inokoshi et al., 2016).

**FIGURE 2:**
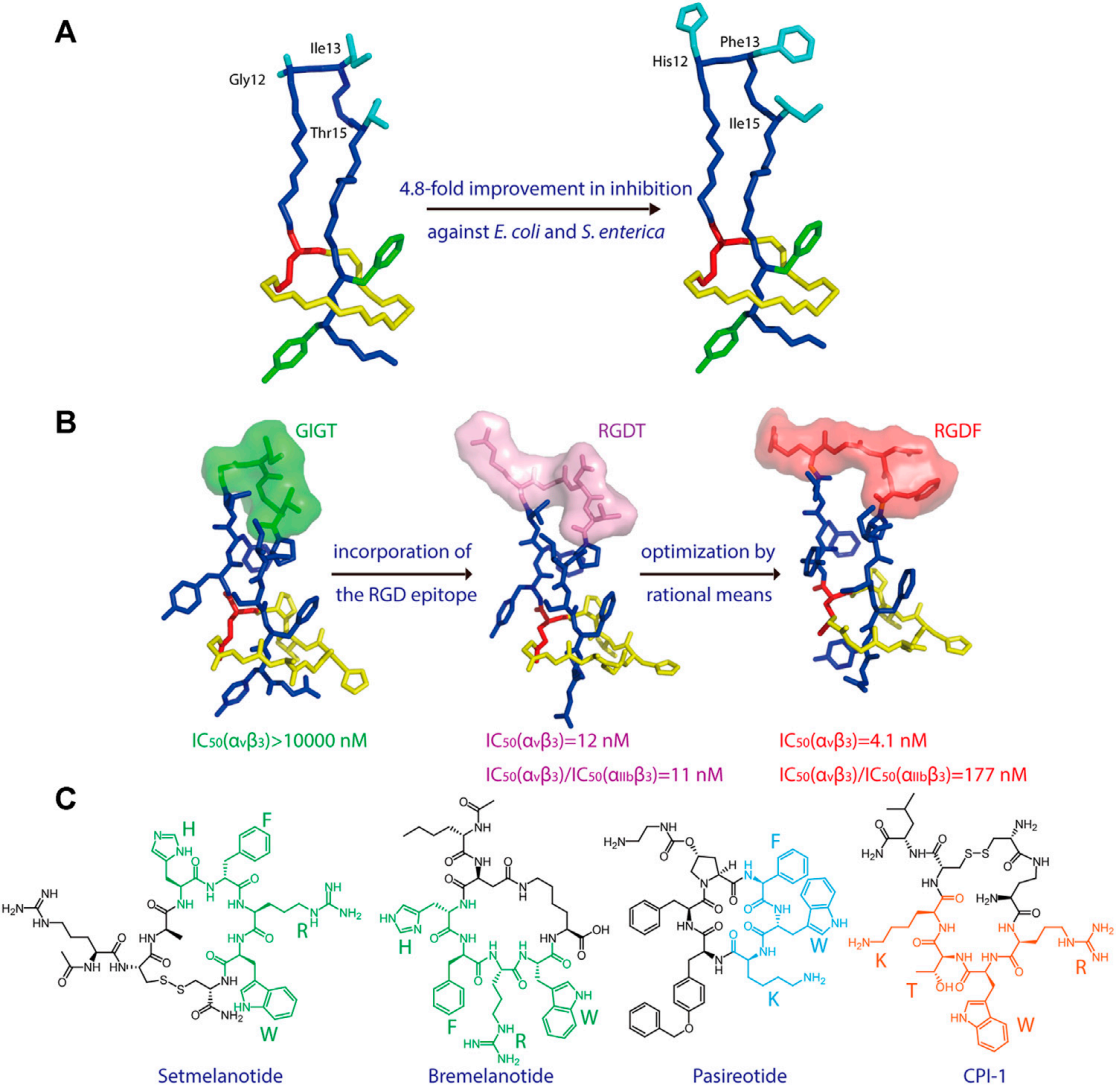
**(A)** Left, NMR structure of MccJ25 based on PDB code 1Q71 (Rosengren et al., 2003). Right, model of a representative MccJ25 variant showing improved inhibitory activity. Residues targeted for mutation are highlighted. **(B)** Epitope grafting of MccJ25. From left to right: NMR structures of wild-type MccJ25 (PDB: 1Q71), MccJ25 RGD (PDB code 2MMW) (Knappe et al., 2011), and MccJ25 RGDF (PDB code 2MMT) (Knappe et al., 2011). The surfaces of the four residues at positions 12–15 are depicted in different colors. **(C)** Peptide drugs containing potential epitopes (highlighted) for lasso peptide engineering. Unless indicated otherwise, lasso peptides are colored as in Figure 1.

Microcin J25 (MccJ25), which specifically inhibits bacterial RNA polymerase activity, is another lasso peptide that has been extensively studied (Yuzenkova et al., 2002; Burkhart et al., 2015; Braffman et al., 2019). The structure-activity relationship of MccJ25 was systematically examined by applying scanning mutagenesis—that is, every possible proteinogenic amino acid was individually introduced at positions 1–7 of the ring and 9–21 of the tail, with one additional Glu-to-Asp substitution at position 8, which supplies the lactam-forming side chain (Pavlova et al., 2008). By quantifying the presence of each of these 381 variants in cell supernatant (via MALDI-MS) and assessing their inhibitory effect on RNA polymerase activity *in vitro* (via fluorescence), the authors clearly defined which residues are indispensable for the biosynthesis and bioactivity, respectively, of the lasso peptide. Interestingly, only a few residues (the lactam-forming Gly1 and Glu8, and the nearby Gly2) were found to be essential for MccJ25 production, indicating broad substrate specificities for the processing enzymes (Pavlova et al., 2008). Only one residue (Tyr9) was shown to be absolutely essential for inhibitory activity *in vitro*, although two other residues in the tail (Phe10 and Phe19) and two in the macrolactam ring (Gly4 and Pro7) and were also deemed important; these same five residues, in addition to Tyr20, were found to be strictly necessary for bacterial cell permeation during *in vivo* growth inhibition assays (Pavlova et al., 2008). Pan and Link developed a system to orthogonally control the expression and transport of MccJ25 in *E. coli* and utilized this system to comprehensively study the structure-activity relationship of the lasso peptide (Pan and Link, 2011). Saturation mutagenesis libraries were constructed while focusing on either the ring or tail segments of MccJ25, leading to the discovery of approximately 100 new MccJ25 analogs that retained antibiotic function (Pan and Link, 2011). Interestingly, multiple-residue substitutions in the tail segment enhanced the potency against MccJ25-sensitive *E. coli* and *Salmonella enterica* strains, with the best analog displaying a 4.8-fold improvement relative to wild-type MccJ25 (Figure 2A). Thus, MccJ25 is considerably tolerant to mutagenesis and could serve as a promising scaffold for therapeutic applications (Pan and Link, 2011).

Recently, Si et al. developed a cell-free biosynthesis (CFB) approach to *in vitro* lasso peptide production (Si et al., 2021). This was accomplished by cloning the respective biosynthetic gene clusters into plasmids and subsequently incubating individual plasmids with crude *E. coli* cell extract and “energy buffer” (i.e., proteinogenic amino acids, NTPs, tRNA, coenzymes, cAMP, 3-phosphoglyceric acid, and spermidine). The lasso peptides burhizin, capistruin, cellulassin, and fusilassin, the single-site variant citrulassin A (D8E), and the predicted lasso peptide halolassin were generated via CFB and confirmed by MS/MS analysis. The authors then applied CFB to the synthesis of a large library of multi-site variants of fusilassin to evaluate the biosynthetic machinery’s substrate tolerance (Si et al., 2021).

Knappe et al. investigated the potential of MccJ25 for epitope grafting, wherein the large loop region of the lasso peptide was substituted with potential recognition epitopes (Knappe et al., 2011). As proof of concept, the integrin-binding motif RGD was grafted onto MccJ25 (Figure 2B), resulting in a notable increase in binding affinity for integrins, with IC_50_ values of 17 (α_v_β_3_), 170 (α_v_β_5_), 855 (α_5_β_1_), and 29.7 nM (α_IIb_β_3_) (Knappe et al., 2011). Hegemann et al. further optimized binding to α_v_β_3_ integrin by preparing an RGDF variant of MccJ25 (Figure 2B) (Hegemann et al., 2014). These results demonstrate that lasso peptides are promising scaffolds for the presentation of functional peptide epitopes.

As peptides account for a substantial proportion of FDA-approved drugs (Al Musaimi et al., 2021), numerous peptide epitopes could potentially be incorporated into lasso peptides to develop new therapeutics (Figure 2C). For example, the two melanocortin receptor agonists setmelanotide and bremelanotide are clinically used to treat obesity and hypoactive sexual desire disorder in women, respectively (Kingsberg et al., 2019; Markham, 2021). The essential “HFRW” sequence in these drugs could possibly serve as an epitope in lasso peptide-type melanocortin receptor agonists (Figure 2C). Another example, the drug pasireotide, was developed to treat the pituitary disorders corticotropinoma and acromegaly (Gomes-Porras et al., 2020). The drug’s key sequence “FWK” may be incorporated into lasso peptides to develop novel somatostatin analogs (Figure 2C). Finally, a cyclic peptide inhibitor CPI-1 targeting the light chain of botulinum neurotoxin A has been reported (Kumaran et al., 2015), and its epitope “RWTK” is a potential candidate for lasso peptide grafting (Figure 2C). Further experiments are needed to test these hypotheses. Given the tolerance of lasso peptides to harsh conditions and amino acid substitutions, the design of lasso peptides bearing active epitopes may help to surmount the shortcomings of peptide-based medicines, such as poor oral bioavailability and proteolytic degradation.

### Design and Assembly of Lasso Peptide Fusion Proteins

Mutagenesis was shown to be an efficient strategy for generating lasso peptide libraries. The screening of such libraries for new functions would benefit from display technology, including bacterial and yeast display, which has emerged as a fundamental approach to detecting peptides with improved properties when combined with fluorescence-activated cell sorting (FACS) (Daugherty 2007). This strategy permits real-time quantification of a fluorescence signal, and thus highly resolved partitioning of peptide libraries, and has already been adopted to improve the properties of several RiPPs. For example, by employing a cyclotide (truncated Agouti-related protein from human) as a scaffold, one study, facilitated by yeast display and FACS, identified ligands that bind α_v_β_3_ integrin with picomolar affinities (Silverman et al., 2009). Another cyclotide, kalata B1, was displayed on the surface of *E. coli* as a scaffold to screen a large knottin (cyclotide-like peptide) library for variants that inhibit human thrombin protease activity (Getz et al., 2011) and antagonize the growth factor receptors neuropilin-1 and neuropilin-2 (Getz et al., 2013). Moreover, yeast and phage display methods have been applied to lanthipeptides to enrich binding affinities for αvβ3 integrin, lipid II, urokinase plasminogen activator, and streptavidin (Urban et al., 2017; Hetrick et al., 2018).

**FIGURE 3:**
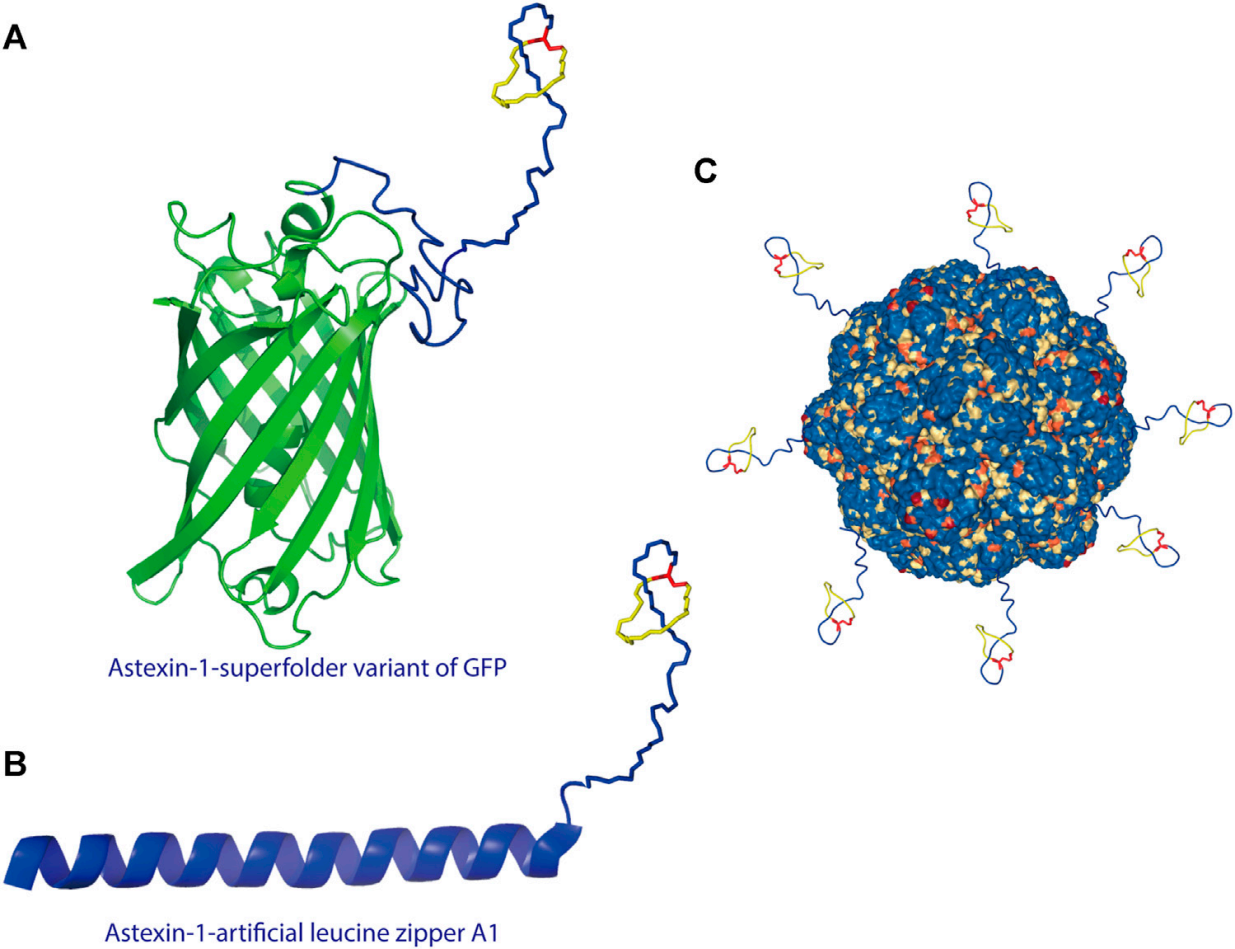
**(A)** Astexin-1 is displayed on superfolder GFP (sfGFP). The (GSSG)_5_-Thb-sfGFP part of the protein (Thb = thrombin cleavage site) was modeled in I-TASSER (Yang and Zhang, 2015) and then linked to the structure of astexin-1 (PDB code 2N68) (Zong et al., 2016). **(B)** Fusion of astexin-1 to the artificial leucine zipper protein A1. Astexin-1 is directly connected to the artificial leucine zipper A1 protein (PDB:4U5T_A). **(C)** Model for functionalization of protein nanoreactors with lasso peptides (PDB:4PT2). Lasso peptides are colored as in Figure 1.

A recent study showed that lasso peptides can also be displayed on scaffold proteins. As proof of concept, the lasso peptide astexin-1 was fused with two model proteins—the superfolder variant of GFP and the artificial leucine zipper A1 (Figures 3A,B) (Zong et al., 2016). Unlike knottins, lasso peptides lack a free N-terminus; thus, they can only be attached via their C-terminus to a scaffold protein. A successful fusion was prepared by introducing a flexible linker between the C-terminal tail of astexin-1 and the N-terminus of either scaffold protein (Zong et al., 2016). This study provided insights into the design of lasso peptide tail fusions that can exhibit augmented functionality. The success of such constructs also offers new approaches for grafting epitopes of interest onto the tail segment of lasso peptides (Zong et al., 2016). Recently, the lasso peptide pandonodin, possessing a particularly long C-terminal tail, was discovered (Cheung-Lee et al., 2019). The authors showed that this tail could be mutated or, like astexin-1, fused to GFP or A1, in some cases without affecting recognition by the processing enzymes. Future searches with phage and bacterial display systems for lasso peptide analogs with novel functions may be possible. Thus, these findings have paved the way for improvements to lasso peptide diversity (Zong et al., 2016; Cheung-Lee et al., 2019).

The successful fusion of a lasso peptide to a protein further provides new perspectives for nanoreactor design. For protein-based nanoreactors designed for drug delivery, a major hurdle is instability due to proteolytic degradation. Recently, Klem et al. attached a knottin mutant (mEETI-II) to TM encapsulin nanoparticles and found that the stable knottins protected the nanocage against tryptic degradation (Klem et al., 2018). On account of their threaded topology, lasso peptides are resistant to proteolysis except by highly specific isopeptidases that cleave the macrolactam ring (Maksimov and Link, 2013; Chekan et al., 2016; Fage et al., 2016). Therefore, from the perspective of design, the display of the lasso peptide on a protein nanocage offers a new approach to heterofunctionalization of protein nanocages (Figure 3C), including specific targeting and stabilization, among other types of functionalization.

### Lasso Peptides With Noncanonical Amino Acids

Due to their ribosomal origin, RiPPs are inherently restricted to the 20 canonical amino acids. However, it is possible to expand the genetic code of microorganisms for the engineering of RiPPs by introducing numerous unnatural or noncanonical amino acids (ncAAs) into the precursor peptide (Budisa, 2013). Using this approach, PTMs can be supplemented with the cotranslational incorporation of ncAAs to considerably diversify the structures and thereby activities of RiPPs (Budisa, 2013). Generally, the following two *in vivo* strategies are available for the introduction of ncAAs into target peptides: supplementation-based incorporation (SPI) and stop-codon suppression (SCS) (Budisa, 2013). In several reports, ncAAs have been incorporated into RiPPs, including lanthipeptides, cyanobactins, and thiopeptides. For example, researchers employed SPI to biosynthesize lanthipeptides with bioorthogonal side chains *in vivo* (Oldach et al., 2012; Kakkar et al., 2018). These side chains can be applied to specific coupling reactions, such as click reactions, to append lanthipeptides with useful chemical moieties (Oldach et al., 2012). In another instance, the SCS approach allowed for incorporation of *p*-chloro-phenylalanine into macrocyclic cyanobactins (Tianero et al., 2012). A similar strategy was used to generate several ncAA-containing thiopeptides, such as thiocillin analogs, which could be further functionalized with fluorophores and photocrosslinkers (Luo et al., 2016).

**FIGURE 4:**
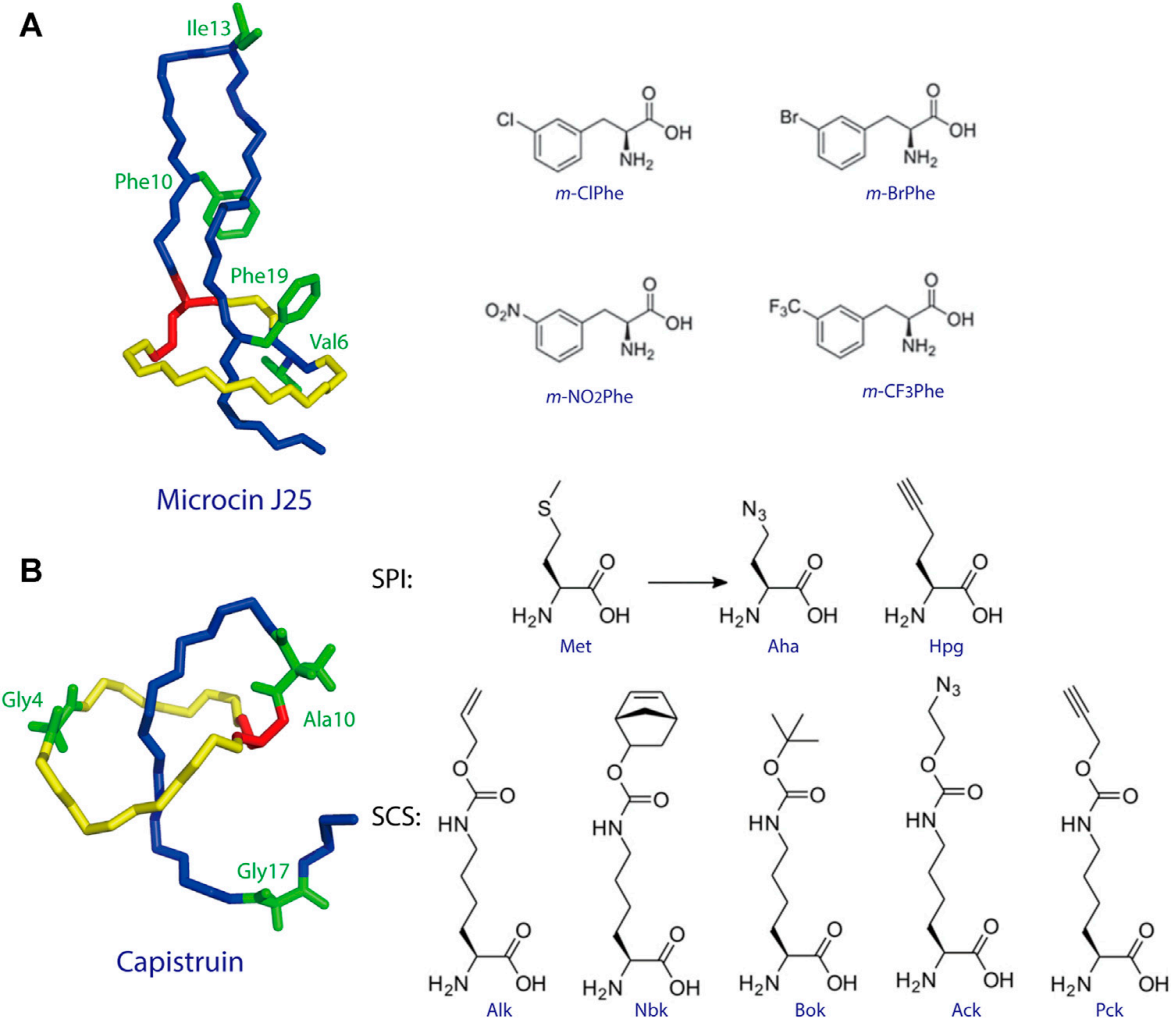
**(A)** Incorporation of ncAAs into MccJ25. The structure of MccJ25 (PDB code 1Q71) (Rosengren et al., 2003) is shown, with positions of incorporation highlighted in green, and the structures of the ncAAs are shown at right. **(B)** Incorporation of ncAAs into capistruin. The structure of capistruin (PDB code 5OQZ) (Jeanne Dit Fouque et al., 2018) is shown, with positions of ncAA incorporation highlighted in green, and the structures of the ncAAs are shown at right. Met = methionine; Aha = azidohomoalanine; Hpg = homopropargylglycine; Nbk = Nε-5-norbornene-2-yloxycarbonyl-l-lysine; Alk = Nε-Alloc-l-lysine; Bok = Nε-Boc-l-lysine; Ack = Nε-2-azidoethyloxycarbonyl-l-lysine; Pck = Nε-2-propyn-1-yloxycarbonyl-l-lysine. Unless indicated otherwise, lasso peptides are colored as in Figure 1.

To date, two studies have demonstrated ncAA incorporation as an effective strategy for tailoring lasso peptides. Piscotta et al. established the feasibility of ncAA incorporation into lasso peptides by introducing four ncAAs at four different positions on MccJ25 (Figure 4A) (Piscotta et al., 2015). The biosynthetic enzymes tolerated all four ncAAs and, in some cases, produced antimicrobial peptides with potency equivalent to that observed for wild-type MccJ25. In another study, both SPI and SCS approaches were applied to the cotranslational introduction of isostructural and orthogonal ncAAs into the lasso peptide capistruin (Figure 4B) (Al Toma et al., 2015). Two ncAAs were incorporated with the SPI method and five with the SCS method. After the ncAAs were inserted, capistruin was further tailored through bioorthogonal chemistry; i.e., the analog Cap_Alk10, containing the ncAA Alk (Nε-Alloc-l-lysine), was reacted with an allyl alcohol (Al Toma et al., 2015). These studies demonstrate that ncAA incorporation is an effective method for not only expanding the chemical and functional space of RiPPs but for probing their biological activities.

Besides these *in vivo* strategies for ncAA incorporation, post-isolation chemical modification of lasso peptides has also been reported. In a recent study, Long et al. reported a strategy for light-activated nitration of tyrosine using bioorthogonal dinitroimidazoles (Long et al., 2021). As proof of concept, the tyrosine plug residues of caulonodin IV were converted to 3-nitrotyrosine, enhancing the lasso peptide’s thermostability and demonstrating that ncAA incorporation is in general an effective approach to introduce bioactive functional groups into lasso peptides. In another example, Allen and Link prepared an MccJ25 variant carrying one Arg and two Cys substitutions and, following cleavage with trypsin after the Arg residue, observed self-assembly of mechanically-interlocked [3] and [4]catenanes between the Cys residues (Allen and Link, 2016). This study provides a new approach for the construction of peptide-based molecular machines.

### C-Terminal Methylation of Lasso Peptides

A more common strategy adopted to diversify the structures of RiPPs relies on modification of the core peptide using characterized processing and tailoring enzymes (Arnison et al., 2013). For proteins, the integration of PTMs into engineering efforts may pose challenges, as the PTM machinery is in general highly sophisticated and must interface with the protein through specific structural features at the region to be functionalized. Conversely, a single leader peptide can guide the introduction of multiple PTMs onto the hypervariable core peptide of a RiPP. For example, only six candidate enzymes were identified for installing the 48 modifications found on marine sponge-derived polytheonamides, including 17 methylations and 18 epimerizations at non-activated carbon centers (Freeman et al., 2012; Morinaka et al., 2014; Freeman et al., 2017; Helf et al., 2019). Highly iterative activities were confirmed for three of these candidates. Ideally, it is possible to exploit processing and tailoring enzymes from different systems by introducing relevant recognition sequences on the leader peptide or core peptide, thereby generating new analogs. For example, van Heel et al. developed a plug-and-play system in which lanthipeptide modification enzymes from different systems were applied to generate novel lanthipeptides with C-terminal aminovinyl-cysteine or d-alanine residues (van Heel et al., 2013). In another study, Burkhart et al. designed leader peptides that could be recognized and processed by enzymes from unrelated RiPP pathways. Through this strategy, hybrid RiPPs were generated using a thiazoline-forming cyclodehydratase and enzymes from the sactipeptide and lanthipeptide biosynthetic pathways (Burkhart et al., 2017). As mentioned above, except for the extraordinary knot structure, further PTMs of lasso peptides were rarely observed until recently. Thus, the discovery of new lasso peptide-tailoring enzymes is necessary for the implementation of this strategy.

**FIGURE 5:**
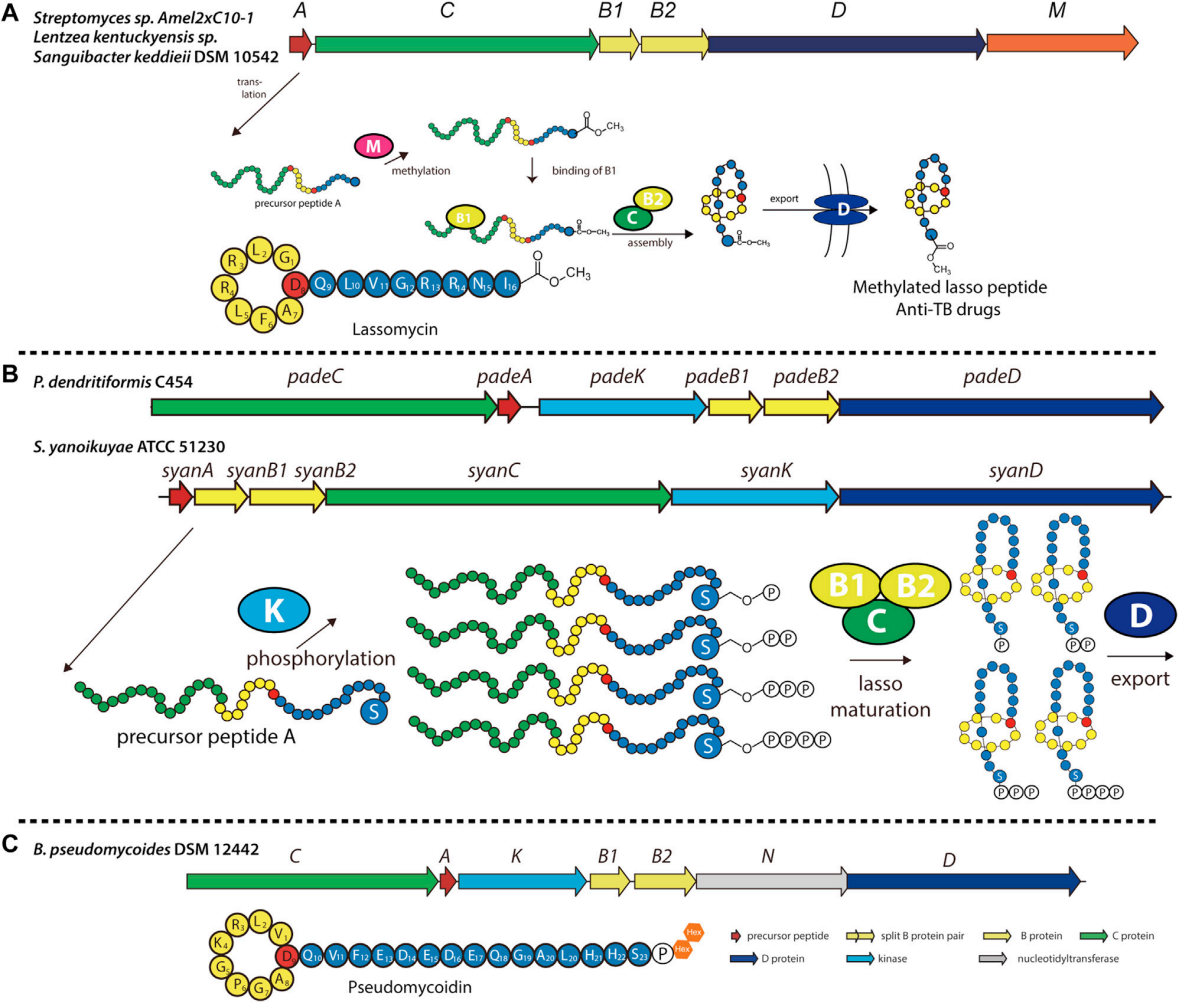
Examples of methylated, phosphorylated, and phosphoglycosylated lasso peptides. **(A)** Biosynthetic gene cluster and pathway for O-methylated lasso peptides, including lassomycin (primary structure schematic shown below). The precursor peptide is modified at its C-terminal carboxyl group by an O-methyltransferase (encoded by gene *M*) prior to lasso folding. **(B)** Biosynthetic gene cluster and pathway for phosphorylated lasso peptides in Firmicutes and Proteobacteria, including paeninodin (from *P. dendritiformis* C454) and a putative lasso peptide (from *S. yanoikuyae* ATCC 51230). The precursor peptide can be phosphorylated multiple times at the hydroxy side chain of its C-terminal Ser by an Hpr kinase homolog (encoded by gene *K*) prior to lasso folding. A circled “P” represents a phosphate group (Zhu et al., 2016b). **(C)** Biosynthetic gene cluster for the phosphoglycosylated lasso peptide pseudomycoidin (primary structure schematic shown below). A kinase (encoded by gene *K*) phosphorylates the hydroxyl side chain of the C-terminal Ser, and a nucleotidyltransferase (encoded by gene *N*) transfers one or two glycosyl groups to that phosphate group. Whether these enzymes act on the precursor peptide or folded lasso peptide is unknown. Lasso peptides are colored as in Figure 1.

Recently, several previously unidentified biosynthetic pathways were discovered and shown to synthesize modified lasso peptides (Montalban-Lopez et al., 2021). Lassomycin, discovered in *Lentzea kentuckyensis* sp. by screening extracts from uncultured species, was found to exhibit promising activity against mycobacteria, including several drug-resistant forms of *M. tuberculosis* (Parish, 2014; Gavrish et al., 2014). While initially thought to adopt a branched-cyclic conformation lacking the lariat knot (Figure 5A) (Gavrish et al., 2014), assays with this chemically synthesized conformer exhibited no biological activity, suggesting that naturally occurring lassomycin is a conventional lasso peptide (Lear et al., 2016). Interestingly, structure elucidation revealed that the C-terminal carboxyl group of lassomycin bears a methyl ester (Figure 5A). Furthermore, whole-genome sequencing showed the biosynthetic gene cluster of lassomycin to be markedly similar to that of the lasso peptide lariatin (Gavrish et al., 2014), with the exception of a putative O-methyltransferase-encoding gene that had not been observed in other lasso peptide gene clusters (Figure 5A). This methyltransferase is likely responsible for installing the methyl ester on lassomycin, either on the precursor peptide or the mature lasso peptide. We recently reported similar methyltransferase-containing gene clusters in *Sanguibacter keddieii* DSM 10542 and *Streptomyces* sp. Amel2xC10-1 (Su et al., 2019). *In vitro* biochemical experiments on StspM from the latter gene cluster demonstrated selective methylation of the C-terminal carboxyl group of precursor peptide substrates with exceptionally broad substrate specificity, making it suitable for combinatorial biosynthesis (Su et al., 2019). As lassomycin was found to bind a negatively charged region of the ClpC1 ATPase complex (Gavrish et al., 2014), C-terminal methylation may play an essential role in recognition (e.g., through charge nullification) and account for germicidal activity. This tailoring enzyme can potentially be exploited to generate other C-terminal methyl esters of lasso peptides to alter or improve their biological functions.

### Phosphorylation, Polyphosphorylation, and Glycosylation of Lasso Peptides in Firmicutes

Previously, all lasso peptides were discovered from either proteobacterial or actinobacterial sources. We recently added Firmicutes to the list by isolating the lasso peptide paeninodin from *Paenibacillus dendritiformis* C454 and thoroughly characterizing its biosynthesis (Zhu et al., 2016c). The paeninodin gene cluster and several others (mainly from *Paenibacillus* and *Bacillus* spp.) were found to contain not only the essential genes for lasso peptide biosynthesis and transport, but an additional gene coding for an HPr kinase homolog (Figure 5B) (Zhu et al., 2016c). *In vitro* and *in vivo* studies demonstrated that the kinase selectively phosphorylates the hydroxyl side chain of the core peptide’s C-terminal Ser; however, the enzyme fails to modify the threaded lasso peptide. Further experiments showed that the kinase specifically recognizes the C-terminal Ser with little specificity for the length or sequence of the rest of the peptide. This nonselective feature renders these kinases useful for applications in combinatorial biosynthesis to generate lasso peptide analogs.

Subsequent genome mining revealed that certain Proteobacteria also contain potential kinase-encoding lasso peptide biosynthetic gene clusters (Zhu et al., 2016b; Zhu et al., 2016c). The organization of these gene clusters was found to differ considerably from those of Firmicutes (Figure 5B). Interestingly, although gene clusters from both phyla encode a kinase, their precursor peptides share little sequence homology except for the presence of a C-terminal Ser, indicating similar substrate specificities for lasso peptide kinases from Proteobacteria and Firmicutes (Zhu et al., 2016b). Indeed, a recent study showed that proteobacterial kinases also phosphorylate the hydroxyl side chain of the C-terminal Ser of their corresponding precursor peptides (Zhu et al., 2016b). Curiously, these kinases incorporated multiple phosphoryl groups at this position on the linear precursor peptide, leading to the generation of polyphosphorylated lasso peptides (Figure 5B) (Zhu et al., 2016b). Thus, this study provides another potential avenue through which lasso peptides can be variegated.

While investigating the novel lasso peptide gene clusters identified from Firmicutes, some were found to contain previously unobserved genes flanking the essential machinery—for example, a putative sulfotransferase-encoding gene located at the start of the gene cluster, and a putative nucleotidyltransferase-encoding gene located between the B2 protease- and D transporter-encoding genes (Zhu et al., 2016c). In several cases, the putative nucleotidyltransferase was fused to the B2 protein, indicating a role for these enzymes in lasso peptide biosynthesis. Indeed, a recent study conducted on one such gene cluster from *Bacillus pseudomycoides* DSM 12442 led to the discovery of pseudomycoidin, a phosphoglycosylated lasso peptide (Figure 5C) (Zyubko et al., 2019). *In vivo* studies showed that the nucleotidyltransferase attaches one or two hexose residues to the phosphorylated lasso peptide (Zyubko et al., 2019), although *in vitro* functionality was not investigated. Whether these enzymes modify precursor peptides (in a manner similar to the HPr kinase homologs) or threaded lasso peptides remains unknown. Studies of these enzymes to elucidate the biological functions of the modified lasso peptides are currently in the pipeline.

### Peptidyl Arginine Deiminase as a Tailoring Enzyme

**FIGURE 6:**
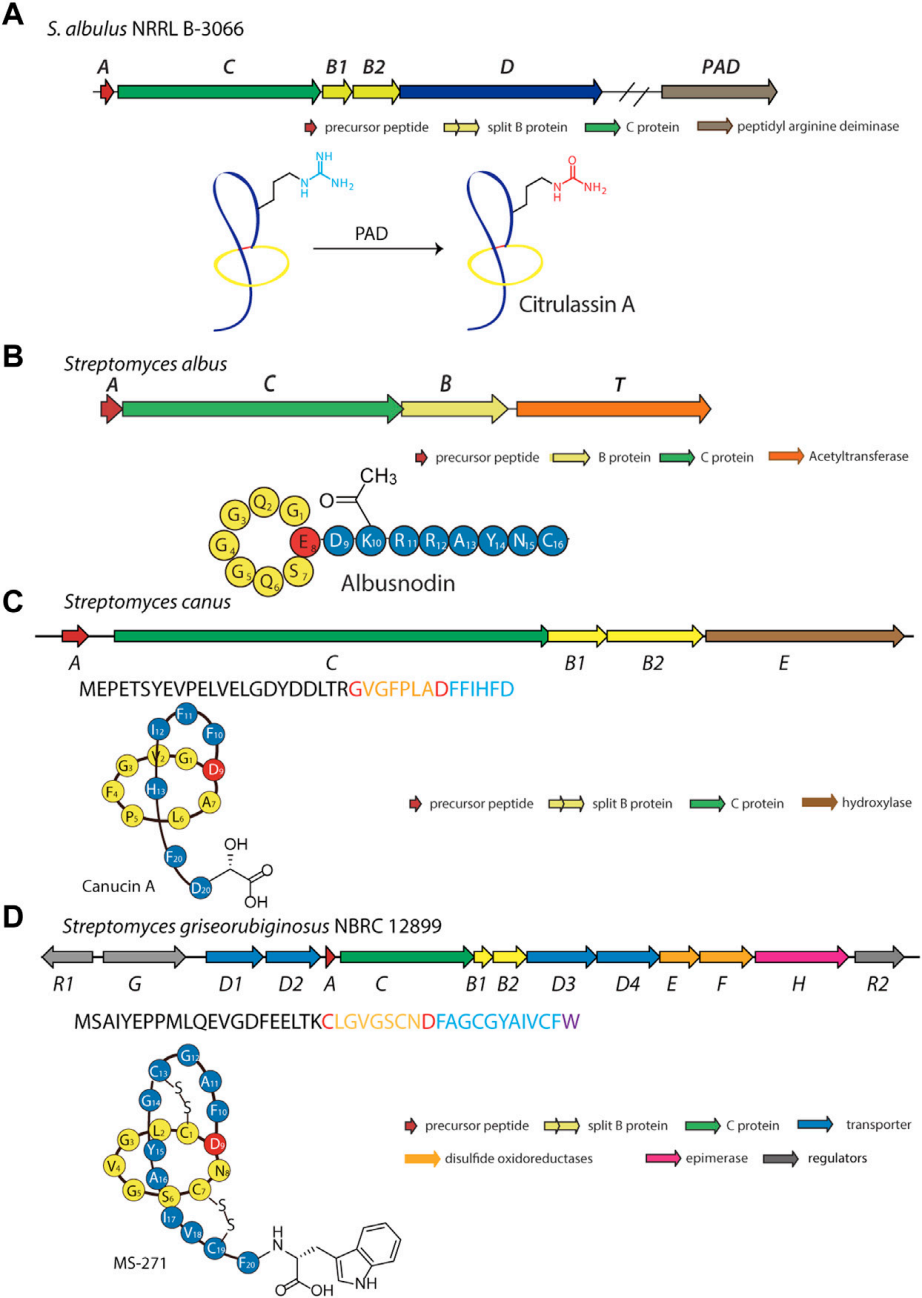
Examples of deiminated, acetylated, hydroxylated, and d-amino acid-containing peptides. **(A)** Biosynthetic gene cluster and pathway for the deiminated lasso peptide citrulassin A. A peptidyl arginine deiminase (PAD) encoded outside the gene cluster is responsible for the citrulline modification. Whether this enzyme acts on the precursor peptide or folded lasso peptide is unknown. **(B)** Biosynthetic gene cluster for the acetylated lasso peptide albusnodin (primary structure schematic shown below). An acetyltransferase (encoded by gene *T*) is responsible for acetylating the primary amine of the side chain of Lys10 prior to lasso folding. **(C)** Biosynthetic gene cluster for the hydroxylated lasso peptide canucin A. An iron/2-oxoglutarate-dependent hydroxylase (encoded by gene *E*) introduces a hydroxyl group on the β-carbon of the C-terminal Asp residue prior to lasso folding. **(D)** Biosynthetic gene cluster for the d-amino acid-containing MS-271 lasso peptide. An epimerase (encoded by the *H* gene) is responsible for inversion of Cα stereochemistry at the C-terminus prior to lasso folding. Lasso peptides are colored as in Figure 1.

In another effort to expand the known members and chemical diversity of the lasso peptide subfamily, Tietz et al. developed RODEO (Rapid ORF Description and Evaluation Online), which combines hidden Markov model-based analysis, heuristic scoring, and machine learning (Tietz et al., 2017). Using this program, the authors identified more than 1,300 systems and isolated six novel lasso peptides, including citrulassin A, which carries an exceedingly rare citrulline modification (Tietz et al., 2017). Nuclear magnetic resonance (NMR) and tandem mass spectrometry (MS/MS) analyses revealed that Arg9 of the lasso peptide was converted to citrulline. To determine the identity of the enzyme responsible for Arg deimination, researchers performed reactivity-based screening with a selective probe for citrulline-containing natural products, leading to the discovery of 13 citrulassin variants (Harris et al., 2020). Interestingly, two distinct *Streptomyces* species (*S. avermitilis* and *S. torulosus*) were found to produce citrulassin variants with identical core peptide sequences, but with the citrulline modification absent in one and present in the other. Comparison of genomic sequences enabled the identification of a potentially responsible peptidylarginine deiminase (PAD), which, unlike other lasso peptide-tailoring enzymes, is not encoded locally in the lasso peptide gene cluster (Harris et al., 2020). *In vivo* complementation experiments confirmed the participation of the PAD (WP_064069847.1) in the biosynthesis of deiminated lasso peptides (Figure 6A). To the best of our knowledge, this is only the second report of PAD activity in the entire bacterial domain (Goulas et al., 2015). Although *in vitro* studies have been performed on the lassomycin and paeninodin tailoring enzymes, none have been performed with this PAD. Moreover, the precise timing of deimination remains mysterious. Future studies are thus necessary to uncover the substrate specificity of this unusual family of enzymes.

### Acetylation of Lasso Peptides

Acetylation is a common type of modification for natural products (Bayer et al., 2004). Using a precursor-centric genome mining approach for lasso peptide discovery, a gene cluster with unique ACBT organization was detected in *Streptomyces albus* DSM 41398 (Figure 6B) (Zong et al., 2018). Here, the *albT* gene encodes a putative acetyltransferase belonging to the GNAT family. BLAST analysis with AlbT as a query enabled researchers to discover 43 similar gene clusters, mainly in Actinobacteria. Through heterologous expression in *Streptomyces coelicolor* and *Streptomyces lividans* hosts, a new lasso peptide bearing an acetyl group was isolated and designated as albusnodin (Zong et al., 2018). Interestingly, *in vivo* studies showed the tailoring step may be essential for lasso peptide folding, thus highlighting the acetyltransferase as a potentially obligate tailoring enzyme.

*In vitro* biochemical characterization of AlbT is yet to be reported; thus, the precise mechanism by which acetylation occurs during albusnodin biosynthesis remains unclear. Because this tailoring step is required for albusnodin production, acetylation may occur prior to formation of the lasso topology, similar to phosphorylation of paeninodin and methylation of lassomycin. The biological function of albusnodin and the specific role of acetylation are yet to be determined; however, the authors proposed that acetylation serves as a resistance mechanism in the producing cells (as demonstrated for other natural products) or aids in folding.

### Hydroxylation of Lasso Peptides

Another potential tailoring enzyme, a hydroxylase, was suggested to modify the RES-701 lasso peptides (Ogawa et al., 1995). MS analysis revealed the only difference between lasso peptides RES-701-1, -2, -3, and -4 to be the tailoring of the C-terminal residue as either Trp (RES-701-1 and -3) or 7-hydroxy-Trp (RES-701-2 and -4). Introduction of this modification during the purification process is unlikely; thus, a specific hydroxylase that can functionalize Trp probably exists. In 2020, a genome mining study of *Streptomyces caniferus* CA-271066 led to the identification of a gene cluster putatively encoding the biosynthetic machinery of RES-701-3 and -4, and production was confirmed through heterologous expression in *S. coelicolor*. ResE, found in the gene cluster and lacking any conserved domains, was proposed to tailor the C-terminal tryptophan of RES-701-3 (Oves-Costales et al., 2020). Future work, including *in vitro* assays, will be necessary to confirm the precise role of this enzyme.

Canucin A is another example of a hydroxylated lasso peptide (Xu et al., 2019). This peptide was discovered in *Streptomyces canus* through a combination of high-throughput elicitor screening and imaging MS, wherein actinomycetes were exposed to a library of natural products for activation of silent gene clusters and subsequent detection of products (Xu et al., 2019). This method uncovered nine novel natural products, including canucin, which carries a β-OH group on the side chain of its C-terminal Asp (Figure 6C) (Xu et al., 2019). *In vitro* studies showed that the *canE* gene product, an iron/2-oxoglutarate-dependent hydroxylase, specifically recognizes the linear precursor peptide of canucin A for modification of the C-terminal residue, similar to other lasso peptide tailoring enzymes (Xu et al., 2019; Zhang and Seyedsayamdost, 2020).

### D-amino Acid-Containing Lasso Peptides

The presence of d-amino acids in peptide natural products is a hallmark of non-ribosomal peptides, which are constructed by NRPS assembly lines (Walsh et al., 2001). Epimerization domains, embedded in many NRPSs, invert the Cα stereochemistry of l-amino acid building blocks for incorporation of d-amino acids into the growing peptide chain (Walsh et al., 2001). Previously, most d-amino acid-containing peptides were thought to be synthesized by NRPSs; however, recent advances have revealed that RiPPs frequently decorate their core peptides with dedicated epimerases (Arnison et al., 2013). For example, the polytheonamides are highly complex 48-mer peptides containing multiple d-configured positions (Freeman et al., 2012). According to metagenomic studies, a single promiscuous enzyme is responsible for modifying up to 18 positions along the peptide (Freeman et al., 2012).

Among all the reported lasso peptides, one d-amino acid-containing peptide, MS-271, was obtained from *Streptomyces* sp. M-271 (Katahira et al., 1996). Recent genome sequencing of the MS-271 producer permitted identification of a lasso peptide gene cluster with a precursor peptide bearing a C-terminal d-Trp (Feng et al., 2018). Heterologous expression of the entire gene cluster in *S. lividans* led to assembly of the mature MS-271 peptide, indicating the presence of a new family of lasso peptide epimerases (Figure 6D) (Feng et al., 2018). Concurrently, a genome mining study discovered another d-amino acid-containing lasso peptide, specialicin, encoded by a similarly organized gene cluster (Kaweewan et al., 2018). Follow-up *in vitro* experiments on the core peptide and full-length precursor peptide demonstrated the *mslH* gene product to be responsible for introducing the d-amino acid (Feng et al., 2021). Similar to the reported lasso peptide methyltransferase (StspM), kinases (ThcoK and SyanK), and iron/2-oxoglutarate-dependent hydroxylase (CanE), MslH specifically recognizes the linear precursor peptide but not the folded lasso peptide, a feature which renders these enzymes useful for peptide engineering.

### Other Potential Tailoring Enzymes for Engineering Lasso Peptides and the Chemoenzymatic Synthesis of Lasso Peptides

Though only a handful of dedicated lasso peptide tailoring enzymes have been experimentally validated, advances in genome mining are expected to unearth further examples. Moreover, as the currently known tailoring enzymes tend to modify precursor peptides, enzymes capable of tailoring folded lasso peptides would add utility to engineering endeavors.

**FIGURE 7:**
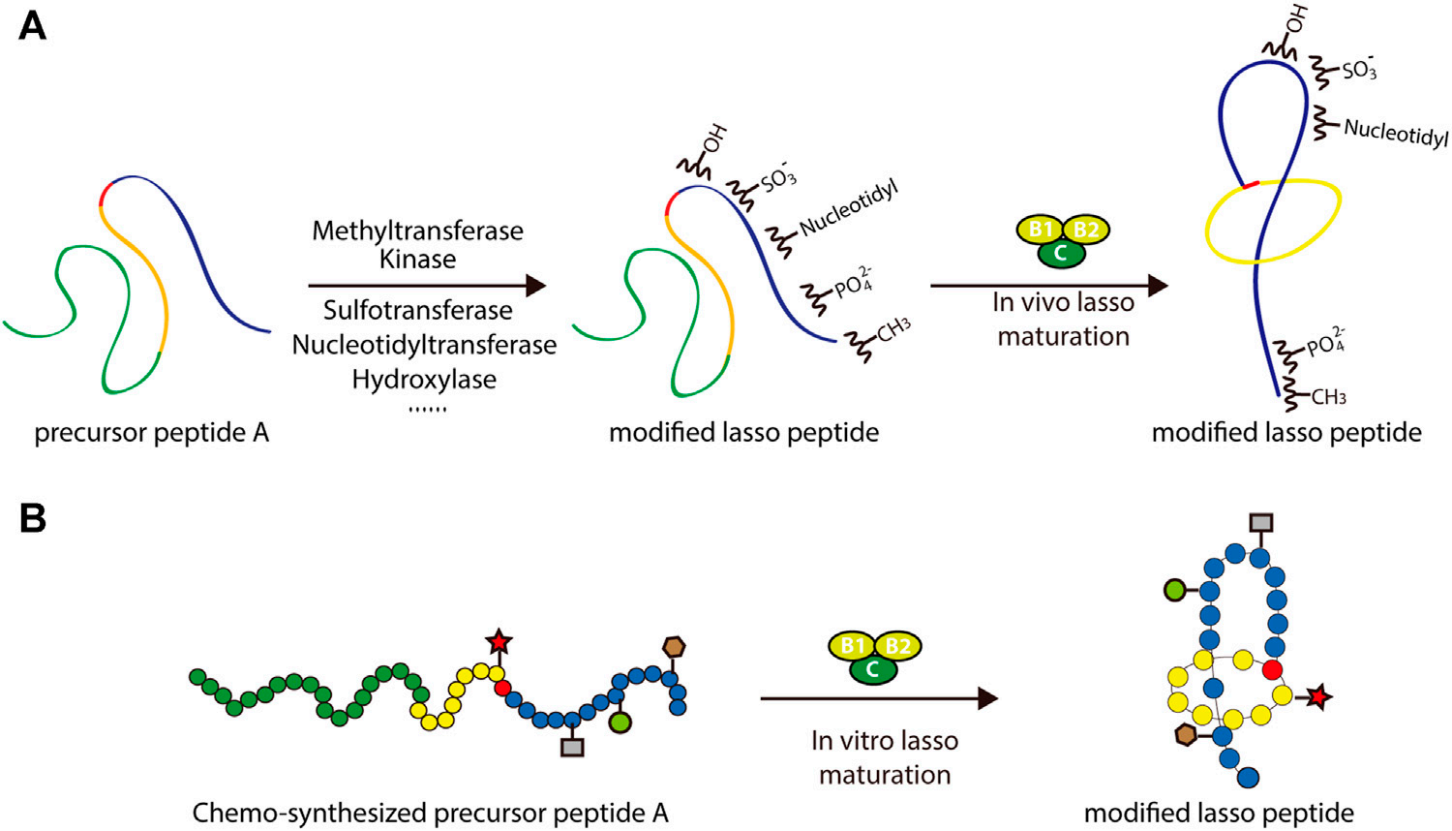
Two approaches to the rational engineering of lasso peptide analogs. **(A)** Co-transformation approach using various tailoring enzymes to introduce new structural features into the lasso peptide scaffold. **(B)** Scheme of chemoenzymatic synthesis of lasso peptide analogs. In both panels, lasso peptides are colored as in Figure 1.

In addition to the enzymes dedicated to lasso peptide maturation, processing enzymes from different subfamilies of RiPPs could be potentially applied to lasso peptides in combinatorial biosynthesis. Recent studies suggest that, in general, RiPP processing enzymes contain structurally conserved precursor peptide-binding domains referred to as RiPP precursor peptide recognition elements (RREs) (Burkhart et al., 2015; Burkhart et al., 2017). By carefully engineering or exchanging these domains, processing enzymes from other RiPP systems could theoretically be used to enhance the chemical functionality of lasso peptides (Figure 7A). Application of tailoring enzymes from other RiPP subfamilies may also be possible, given adequate promiscuity toward the core peptide of interest.

An alternative approach to this end involves *in vitro* chemoenzymatic synthesis or *in vitro* mutagenesis. Incubating biosynthetic enzymes with chemically synthesized substrate analogs, such as those including nonproteinogenic amino acids, is an effective way of expanding the chemical space of peptide natural products. Several successful examples of this approach can be cited. By incubating chemically synthesized substrates with the lacticin 481 biosynthetic machinery, Levengood et al. prepared ncAA-containing lantibiotic analogs, several of which displayed improved antimicrobial activity (Levengood et al., 2009). Another example is the derivation of microviridin peptides, which exhibit an unusual architecture featuring non-canonical lactone and lactam rings. In a study by Reyna-González et al., an efficient *in vitro* mutasynthesis system for microviridins was developed based on the use of two ATP-grasp ligases that were constitutively activated through covalent attachment of the leader peptides to a GNAT-type N-acetyltransferase (Reyna-Gonzalez et al., 2016). This approach facilitated the *in vitro* chemoenzymatic synthesis of microviridin variants capable of targeting serine proteases, namely trypsin and subtilisin. Such examples frame *in vitro* chemoenzymatic synthesis as a viable method for diversifying RiPPs and modulating their bioactivities (Figure 7B).

Recently, several groups have reported the *in vitro* mutagenesis of lasso peptides (Yan et al., 2012; Burkhart et al., 2015; Assrir et al., 2016; Zhu et al., 2016a; Cheung et al., 2016; DiCaprio et al., 2019; Koos and Link, 2019; Si et al., 2021). In a foundational study, the biosynthesis of MccJ25 was reconstituted *in vitro* by incubating the precursor peptide McjA with processing enzymes McjB and McjC in the presence of ATP and Mg^2+^ (Yan et al., 2012). This confirmed the individual functions of the processing enzymes while highlighting their interdependence: McjB is responsible for leader peptide cleavage and McjC for macrolactam ring formation, with the two enzymes potentially forming an interaction complex for lasso peptide biosynthesis. By adopting this *in vitro* system, MccJ25 analogs were generated. Knowledge of lasso peptide biosynthetic systems featuring split B proteins was also advanced through *in vitro* studies. The B1 proteins from the streptomonomicin, lariatin, and paeninodin lasso peptide systems were found to specifically bind to the leader sequences of their precursor peptides (Burkhart et al., 2015; Zhu et al., 2016a; Cheung et al., 2016; Hegemann et al., 2018). The B1 protein is a standalone RRE protein, which exhibits a conserved structure (Sumida et al., 2019). Further research has shown that the B1 protein first binds to the leader peptide and then delivers its peptide substrate to the B2 protein for processing (Zhu et al., 2016a; Hegemann et al., 2018). More recently, two groups reported the *in vitro* synthesis of fuscanodin or fusilassin from *Thermobifida fusca* (using proteins heterologously expressed in and purified from *E. coli*), thus providing a new platform to access assorted analogs (e.g., a lasso peptide with a ten-residue macrolactam ring) (DiCaprio et al., 2019; Koos and Link, 2019). The recent platform for cell-free lasso peptide biosynthesis established by Si et al. (described above) further expands upon this potential (Si et al., 2021). These studies lay the groundwork for *in vitro* mutagenesis of lasso peptides. Ideally, by merging rationally designed peptide-binding domains with B2 and C proteins, the logical or combinatorial generation of modified lasso peptides is achievable (Figure 7B).

### Lasso Peptides as a Potential Scaffold for Hybrid Catalyst

**FIGURE 8:**
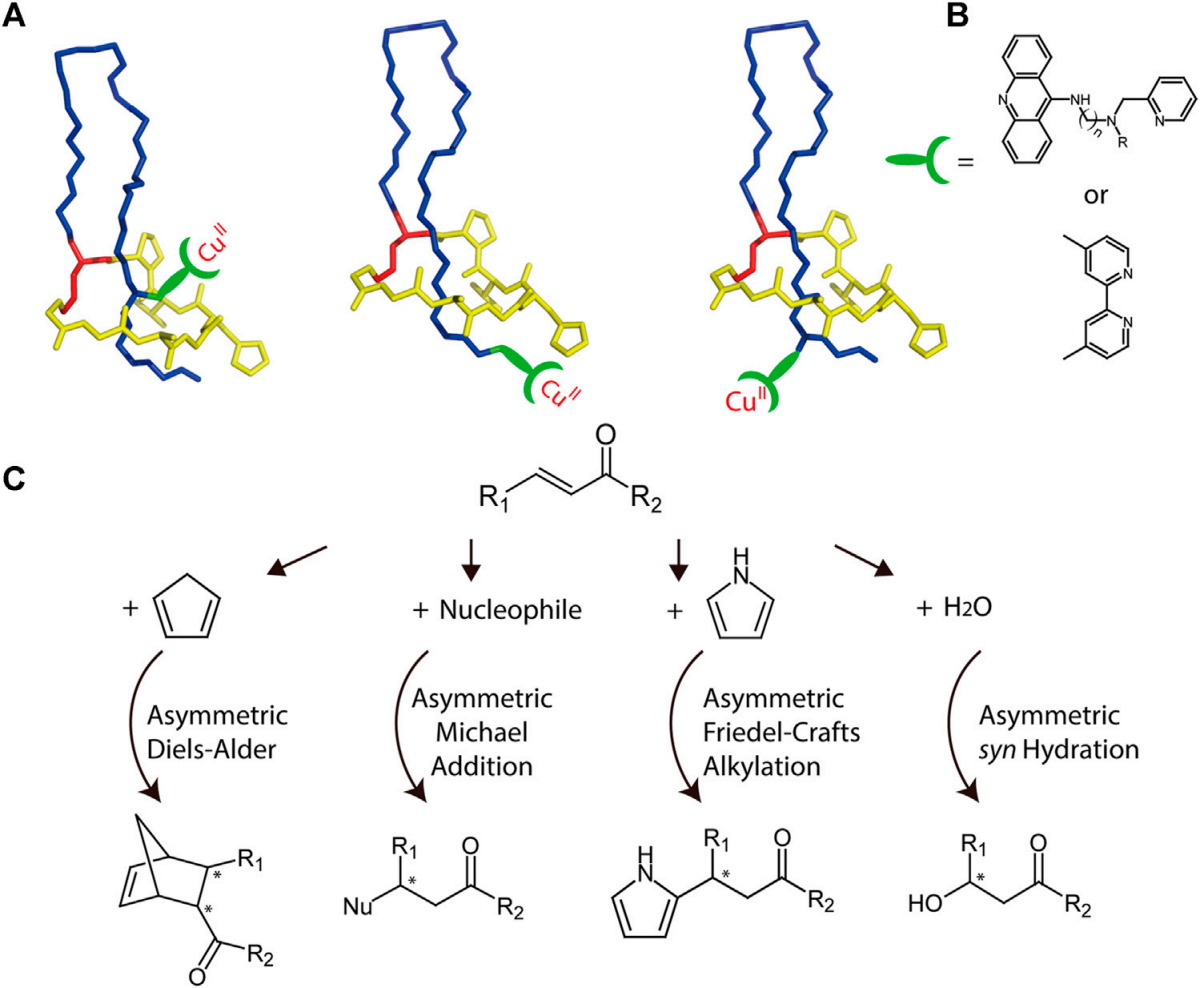
**(A)** Schematic representation of potential MccJ25-based asymmetric catalysis using anchoring strategies. The anchoring site should be subjected to optimization. Lasso peptides are colored as in Figure 1. **(B)** Two potential ligands that may be used for transition metal binding. **(C)** Potential asymmetric reactions that may be facilitated by the hybrid catalyst.

Biopolymers with chiral architectures, such as proteins and DNA, are ideal ligands for stereoselective catalysis in organocatalytic applications (Deuss et al., 2011). This type of hybrid catalyst can combine transition metal chemistry with ligand-functionalized biopolymers to facilitate reactions not observed in nature. A variety of highly enantioselective reactions have been reported through application of such catalysts (Boersma et al., 2010; Megens and Roelfes, 2011; Rioz-Martínez and Roelfes, 2015). The chiral microenvironment and second coordination sphere interactions furnished by biopolymers are crucial for achieving high enantioselectivity and accelerated catalysis, and the well-characterized lasso structure provides multiple potential chiral microenvironments for achieving optimal catalyst design (Figure 8). For example, transition metals can be attached either above or below the ring, and peptide variants can be readily prepared through mutagenesis to further alter the chiral microenvironments. Collectively, these features render lasso peptides suitable for engineering sophisticated catalysts.

As a potential example, copper complexes are considered as covalently bonded to lasso peptides (Figures 8A,B). Previously, by combining various copper dinitrogen complexes with commercially available DNA, scientists designed several catalysts to perform asymmetric Diels–Alder, Michael addition, and Friedel–Crafts alkylation reactions with high enantiomeric excess values (Rioz-Martínez and Roelfes, 2015). Using a similar approach, lasso peptides may also be engineered to prepare highly efficient metalloenzymes, though this potential must be investigated experimentally (Figure 8C).

## Conclusion and Outlook

Lasso peptides are a growing subfamily of RiPP natural products that possess a notable knot structure (Maksimov et al., 2012; Hegemann et al., 2015). This unusual fold confers resistance against thermal, proteolytic, and chemical treatment, and enables a wide spectrum of bioactivities, including antimicrobial, antiviral, enzyme inhibitory, receptor antagonist, and antitumor activities (Maksimov et al., 2012; Hegemann et al., 2015; Cheng and Hua, 2020). However, this topology also poses challenges for chemical synthesis approaches to lasso peptide design (to date, only one successful total synthesis of a folded lasso peptide has been reported, with a limited yield of 2.5%) (Chen et al., 2019). Thus, the ability to generate diverse lasso peptide analogs chemically is currently limited.

Numerous advancements, mostly over the past decade, have facilitated the study of lasso peptides. The combined thermal-proteolytic stability assay, wherein the lasso peptide is incubated at elevated temperatures and then subjected to proteolytic cleavage followed by LC-MS/MS analysis, has been widely implemented as a simple and reliable method of predicting lasso topologies without relying on NMR spectroscopy or X-ray crystallography (Hegemann, 2020). More recently, MS fragmentation methods like electron-capture dissociation (ECD), collision-induced dissociation (CID), and electron-transfer dissociation (ETD) and MS separation methods like ion mobility-mass spectrometry (IM-MS) have permitted the rapid prediction of lasso conformations from minute quantities or mixtures (Zirah et al., 2011; Pérot-Taillandier et al., 2012; Jeanne Dit Fouque et al., 2015; Jeanne Dit Fouque et al., 2018). Given the ribosomal origins and promiscuous processing enzymes of lasso peptide precursors, sizeable libraries of lasso peptides can be readily prepared via mutagenesis, particularly when coupled with the recently designed cell-free biosynthesis strategy for lasso peptide production (Maksimov et al., 2012; Hegemann et al., 2015; Si et al., 2021). To circumvent poorly expressing or silent lasso peptide biosynthetic gene clusters, as well as genetically intractable or unculturable hosts, heterologous expression systems (e.g., *E. coli*, *Streptomyces,* and *Burkholderia* hosts) have been established to produce lasso peptides from Actinobacteria and Proteobacteria; nevertheless, identification of further suitable hosts for production of lasso peptides from other phyla would be advantageous (Hegemann et al., 2013b; Li et al., 2015; Sánchez-Hidalgo et al., 2020). The recent application of high-throughput elicitor screening with imaging MS to lasso peptides is also anticipated to bypass these hurdles (Xu et al., 2019). Lastly, bioinformatics approaches have dramatically expanded the known varieties of lasso peptides and have resulted in the discovery of previously unknown tailoring enzymes that can be used to add functionality to an array of lasso peptides (Maksimov et al., 2012; Hegemann et al., 2015; Zhu et al., 2016b; Zhu et al., 2016c; Tietz et al., 2017; Kaweewan et al., 2018; Zong et al., 2018; Oves-Costales et al., 2020).

Despite the aforementioned achievements, significant obstacles still limit the success and application of lasso peptide research. The majority of lasso peptides are underexploited in biotechnology, medicine, and agriculture, in some cases because their potentially valuable activities have gone undetected (an obstacle compounded by the fact that the biological function of most lasso peptides in nature remains cryptic) (Hegemann et al., 2015). Adaptation of high-throughput, activity-driven assays to lasso peptides, especially in combination with display technology, is thus desirable. Lasso peptides have also been underexploited because of yield constraints. As only one lasso peptide has been chemically synthesized, production of lasso peptides relies on the overexpression of active processing enzymes (Chen et al., 2019). The cell-free biosynthesis strategy for lasso peptides may help to overcome limitations in scalability, although processing enzymes from additional organisms will need to be implemented if a wider range of peptide sequences is to be processed (Si et al., 2021). In this regard, three-dimensional structures of B2 and C proteins, particularly in complex with a peptide substrate, would aid in establishing general rules for the substrate tolerance and specificity of these processing enzymes. Likewise, the elements of recognition employed by lasso peptide tailoring enzymes are poorly understood; a firmer grasp of these elements could, for example, enable the engineering of enzymes from other RiPP subfamilies to target and modify lasso peptides.

Interestingly, the company Lassogen was founded in 2019 with the goal of designing lasso peptide-based drugs for cancer treatment. Two of Lassogen’s drug candidates, LAS-103 and LAS-20x, which increase the immune response to endothelin receptor type B and limit chemokine receptor CCR4-based immune suppression, respectively, are currently under development (Lassogen, 2021). These compounds represent the growing interest and future promise of lasso peptide-derived therapeutics.

Finally, the use of lasso peptides as special scaffolds, which is not limited to therapeutics, is expected to garner more attention in the future. For example, a hybrid catalyst may be designed through rational engineering approaches or bioconjugate chemistry principles. In summary, the strategies discussed herein provide ample opportunities to design novel, chemically enriched lasso peptide analogs that may possess valuable functionalities.
